# Biodegradation of Phenanthrene and Heavy Metal Removal by Acid-Tolerant *Burkholderia fungorum* FM-2

**DOI:** 10.3389/fmicb.2019.00408

**Published:** 2019-03-14

**Authors:** Xin-xin Liu, Xin Hu, Yue Cao, Wen-jing Pang, Jin-yu Huang, Peng Guo, Lei Huang

**Affiliations:** Tianjin Key Laboratory of Organic Solar Cells and Photochemical Conversion, College of Chemistry and Chemical Engineering, Tianjin University of Technology, Tianjin, China

**Keywords:** phenanthrene biodegradation, *Burkholderia fungorum*, heavy metal resistance, acid-tolerant bacteria, bioremediation

## Abstract

Phenanthrene (PHE) is a common pollutant of acidic and non-acidic environments that is recalcitrant to biodegradation. Herein, *Burkholderia fungorum* FM-2 (GenBank accession no. KM263605) was isolated from oil-contaminated soil in Xinjiang and characterized morphologically, physiologically, and phylogenetically. Environmental parameters including PHE concentration, pH, temperature, and salinity were optimized, and heavy metal tolerance was investigated. The MIC of strain FM-2 tolerant to Pb(II) and Cd(II) was 50 and 400 mg L^−1^, respectively, while the MIC of Zn(II) was >1,200 mg L^−1^. Atypically for a *B. fungorum* strain, FM-2 utilized PHE (300 mg L^−1^) as a sole carbon source over a wide pH range (between pH 3 and 9). PHE and heavy metal metabolism were assessed using gas chromatography (GC), inductively coupled plasma optical emission spectroscopy (ICP-OES), scanning electron microscopy-energy dispersive X-ray spectroscopy (SEM-EDS), Fourier-transform infrared (FTIR) spectroscopy and ultraviolet (UV) absorption spectrometry. The effects of heavy metals on the bioremediation of PHE in soil were investigated, and the findings suggest that FM-2 has potential for combined bioremediation of soils co-contaminated with PHE and heavy metals.

## Introduction

Polycyclic aromatic hydrocarbons (PAHs) fused-ring aromatic compounds consisting of two or more fused benzene and/or pentacyclic rings (Ghosal et al., 2016; Baltrons et al., 2018). PAHs are frequently found alongside heavy metals in contaminated environments including manufacturing plants and refinery sites, and the presence of heavy metals may interfere with PAH biodegradation (Murthy et al., 2014; Quero et al., 2015; Su et al., 2018). Biodegradation is considered an efficient, economic, and facile physicochemical process for the treatment of environments contaminated with PAHs (Mnif et al., 2017; Gong et al., 2018). Novel bacterial strains isolated from soils contaminated with mixtures of pollutants may possess sophisticated catabolic capabilities and the ability to tolerate, extreme conditions, toxic metals, and limited nutrients (Oves et al., 2012; Rashid et al., 2016). Such organisms that can also degrade PAHs may offer an effective and affordable remediation strategy (Kuppusamy et al., 2016).

Members of the *Burkholderia* genus play significant ecological roles and hold potential for biotechnological applications. Species from the plant-beneficial-environmental (PBE) *Burkholderia* cluster are able to exploit diverse aromatic compounds as sources of energy and carbon, and some have considerable biotechnological potential due to their ability to degrade chemical pollutants (Suarez-Moreno et al., 2012). Strains of *B. xenovorans, B. vietnamiensis, B. fungorum, B. kururiensis, B. unamae, B. sartisoli*, and *B. phenoliruptrix* are employed for bioremediation of polluted environments because they are able to tolerate and metabolize compounds that are recalcitrant to degradation (Caballero-Mellado et al., 2004, 2007; Coenye et al., 2004; O'Sullivan and Mahenthiralingam, 2005; Vanlaere et al., 2008; Felice et al., 2016). However, there are few reports of strains belonging to *Burkholderia* that can tolerate heavy metals and PAHs (Kuppusamy et al., 2016).

In the present work, the PAH-degrading FM-2 strain was isolated from oil-contaminated soils in an oil field in Xinjiang, and classified as *Burkholderia fungorum* based on phenotypic and phylogenetic analyses. PHE was selected as a model PAH because it is a widespread pollutant with typical PAH characteristics including a K region and a bent structure. Unusually for a *B. fungorum* strain, FM-2 can degrade PHE over a wide pH range, including under highly acidic conditions. Herein, we investigated the effectiveness of the PAH-degrading FM-2 for remediating soils co-contaminated with heavy metals and PHE.

## Materials and Methods

### Sampling, Chemicals, and Culture Media

Samples were collected from oil-contaminated soil in Xinjiang oilfield (Xinjiang, China). Bacterial strains capable of degrading PHE were isolated using the selective enrichment method. PHE (purity ≥97%) and other reagents were purchased from Energy Chemical Technology Co. Ltd.

Minimal medium (pH 7 ± 0.2) consisting of 0.1 g MgSO_4_, 2.04 g KH_2_PO_4_, 12.5 g Na_2_HPO_4_·12H_2_O, and 0.4 g (NH_4_)_2_SO_4_ (per L of distilled water) was used for isolating microorganisms capable of utilizing PHE as a sole source of carbon and energy source.

Enrichment of the strain before screening was performed using mineral salt medium (pH 7 ± 0.2) containing 0.7 g MgSO_4_, 3.48 g KH_2_PO_4_, 1.5 g Na_2_HPO_4_·12H_2_O, 3.96 g (NH_4_)_2_SO_4_, and 0.01 g yeast per L of distilled water.

LMM medium (pH 6.5) contained 0.1 g KH_2_PO_4_, 0.1 g Na_2_HPO_4_, 0.5 g NH_4_NO_3_, 0.5 g (NH_4_)_2_SO_4_, 0.2 g MgSO_4_, 20 mg CaCl_2_, 2 mg FeCl_2_, and 2 mg MnSO_4_ per L of distilled water (Ramadass et al., 2016).

### Enrichment Isolation and Morphological and Biochemical Characterization

Screening of conditions for PHE-degrading strains was performed as described previously (Mnif et al., 2017), and the isolated FM-2 strain was selected for phylogenetic analysis. Typical biochemical and physiological characteristics were systematically evaluated as described in the manual of common bacterial identification (Zhu and Ying, 2001).

### PHE Degradation Test

FM-2 was selected using dilution plates based on obvious colony morphology, and transferred to fresh lysogeny broth (LB) agar plates several times to guarantee culture purity. A single colony was picked from the final LB agar plate and inoculated in 30 mL mineral salt medium with 2% glycerol. After culturing for 48 h at 200 rpm and 25°C, a 600 μL aliquot of the fermentation broth was transferred to 30 mL minimal medium containing PHE (300 mg L^−1^). PHE was dissolved in n-hexane, and then added into 100 ml flask at final concentration of 300–600 mg L^−1^. The flasks were shaken for 2 h at 200 rpm and 25°C. A thin film of PHE was left at the bottom of the flask after removing n-hexane, and then MM was added. The enrichment culture was sub-cultured aerobically (shaking at 200 rpm) at 25°C to facilitate degradation of PHE. Bacterial cell growth was monitored by measuring the absorbance (Abs) at 600 nm, and degradation of PHE was confirmed by both color change and gas chromatography (GC) analyses. The remaining PHE was extracted from the whole culture (30 mL) in a erlenmeyer flask twice with 30 ml of n-hexane (>98%, benchmark, China). The organic phases were combined and dried with anhydrous Na_2_SO_4_. The solvents were removed under reduced pressure and the residues were dissolved in n-hexane. Gas chromatograph equipment (BRUKER 456-GC, USA) was applied to detect the variation of PHE in the n-hexane. Ten microliter of the organic phase was analyzed by using a HP-5 type capillary column (30-m length × 0.32-mm ID, 0.25-μm film thickness). During the analytical process, the column temperature was kept at 80°C for 1 min, then programmed to 290°C at a rate of 10°C min^−1^ which was maintained for 10 min. Media not inoculated with cells were used as sterile control. Control tests with different PHE concentrations were performed to evaluate the actual PHE recovery efficiency after extraction, which was over 87.2% at 300–600 mg L^−1^ PHE. The above mentioned methods are based on the approach reported by Tao et al. (2007), similar with the determinating technique of PHE degradation rate (Muangchinda et al., 2013; Oyetibo et al., 2017; Reddy et al., 2017).

$$\begin{array}{l} \text{PHE degradation rate}=(C_{1}-D_{2})/C_{1}\times 100 \end{array}$$

*C*_1_ - PHE recovery rate in controled experiment after *t* d, %; *D*_2_ - PHE recovery rate in degradation experiment after *t* d, %.

### 16S rRNA Gene Sequence Determination and Phylogenetic Analysis

Analysis of the 16S rRNA gene was performed as described in our previous work (Huang et al., 2013), and the nucleotide sequence of the gene in FM-2 has been deposited in GenBank under accession number KM263605.

### Determination of PHE Degradation Rate and Optimisation of Biodegradation

The effect of PHE concentration on the growth of FM-2 was assessed in 100 mL Erlenmeyer flasks containing 30 mL minimal medium (2% FM-2, v/v) supplemented with 300, 400, 500, and 600 mg L^−1^ PHE as a sole carbon source. Flasks were incubated for 3 days at 25°C on rotary shaker (200 rpm), and the initial pH was adjusted to 7.0. A control culture inoculated with boiled FM-2 was also included, and PHE degradation was evaluated by measuring the change in absorbance at 600 nm, and also by GC analysis (Tao et al., 2007).

The pH, salinity, temperature, and cultivation duration were optimized using an appropriate amount of biomass in a 100 mL conical flask containing 30 mL minimal medium and 300 mg L^−1^ PHE. The pH was tested at intervals of 1.0 between 2.0 and 11.0. NaCl was tested between 0 and 20 g L^−1^ in minimal medium at pH 7.0, the temperature was assessed between 15 and 40°C, cultivation was performed for 1, 2, and 3 days, and the inoculation volume and sampling operations were as described above.

### Effect of Heavy Metal Ions

Heavy metal resistance of the PHE-degrading FM-2 strain was assessed using 20 mg L^−1^ ZnSO_4_·7H_2_O, Pb(NO_3_)_2_, and CdCl_2_·5/2H_2_O based on minimum inhibitory concentration (MIC) tests, and working test solutions were sterilized filtration trough a 0.45 μm membrane. LMM medium was separately supplemented with each of the heavy metals and 300 mg L^−1^ PHE, inoculated with 2% (v/v) FM-2, and cultured at 25°C with shaking at 200 rpm for 7 days. Cultures containing LMM, FM-2, and each heavy metal but without PHE were included as control I, and LMM supplemented with FM-2 and 300 mg L^−1^ PHE but without heavy metals were included as control II. Finally, a sterile control culture without FM-2 was included to assess abiotic loss of PHE.

### Analytical Tests

#### Analysis of the Bioaccumulation of Heavy Metal in FM-2 Cells by Inductively Coupled Plasma Optical Emission Spectroscopy (ICP-OES) and Adsorption Isotherms

Bioaccumulation of heavy metals was evaluated by inoculating *B. fungorum* FM-2 into LMM medium containing 200 mg L^−1^ Zn (II), 50 mg L^−1^ Pb (II), or 200 mg L^−1^ Cd(II) as well as 300 mg L^−1^ PHE. Cultures were incubated at 25°C with shaking at 200 rpm for 1, 3, 5, and 7 days. As described previously (Chiboub et al., 2016) with some modifications, cultures were centrifuged (8,000 rpm for 5 min at 4°C) and pellets were washed three times with sterile distilled water to remove free heavy metal ions. Pellets were then treated with 10 mM sterile EDTA at 25°C with agitation at 200 rpm for 30 min. Following centrifugation (8,000 rpm for 10 min at 4°C), the pellet and supernatant (S_1_) were separated in different 50 mL centrifuge tubes, pellets were washed three times with sterile distilled water, disrupted using an ultrasonic cell disrupter, and the resulting supernatant (S_2_) was collected by centrifugation (10,000 rpm for 20 min at 4°C). S_1_ and S_2_ were analyzed using a Vista MPX ICP-OES instrument (Varian, Palo Alto, CA) to determine the heavy metal content of cell walls (S_1_) and the intracellular space (S_2_) (Wei et al., 2009).

Adsorption isotherms was determined by the following experiment: strain FM-2 was inoculated into LMM medium containing 200 mg L^−1^ Zn (II), 50 mg L^−1^ Pb (II), or 200 mg L^−1^ Cd(II) as well as 300 mg L^−1^ PHE. Cultures were incubated at 25°C with shaking at 200 rpm for 0, 1/2, 1, 2, 3, 4, 5, 6, and 7 days. Specific experiment procedures were carried out according to the literature reported by Vishan et al. (2017b).

#### Scanning Electron Microscopy-Energy Dispersive X-ray Spectroscopy (SEM/EDS)

PHE-degrading *B. fungorum* FM-2 was cultured in LMM containing 200 mg L^−1^ Zn(II), 50 mg L^−1^ Pb (II), or 200 mg L^−1^ Cd(II) at 25°C with shaking at 200 rpm for 7 days as described previously (Kang et al., 2014), and a SUPRA 35 VP field emission SEM instrument combined with an X-Max EDS instrument (Oxford Instruments) were used to analyse the surface morphology cells following biosorption of Zn (II), Pb (II), and Cd (II).

#### Fourier-Transform Infrared Spectroscopy (FTIR)

For FTIR analyses, strain FM-2 was cultured as described in Section Analysis of the Bioaccumulation of Heavy Metal in FM-2 Cells by Inductively Coupled Plasma Optical Emission Spectroscopy (ICP-OES) and Adsorption Isotherms, cell pellets were obtained by centrifugation (8,000 rpm, 10 min, 4°C), washed three times with deionised water, and lyophilized. Bacterial cells grown in LMM without metal ions served as a control. FTIR spectra of KBr pellets of dried cells associated with or without Zn (II), Pb (II), and Cd (II) were obtained using a Vector 33 FTIR instrument (Bruker, Germany).

### Bioremediation of Soil Co-contaminated With Heavy Metal Ions and PHE

#### Soil and Preparation

Topsoil in which PAHs were not detectable was collected from an agricultural field in Xiqing District, Tianjin, China. After transfer to the laboratory, soil was air-dried, and sieved through a 2 mm mesh. Incorperate 10 mg kg^−1^ zinc sulfate or 6 mg kg^−1^ cadmium chloride into soils, respectively. All cadmium and zinc amendments were achieved by dissolving 200 mg ZnSO_4_·7H_2_O or CdCl_2_·5/2H_2_O in 10 mL of sterile deionised water prior to adding to the appropriate soil treatments. Then PHE was mixed with soil at 150 mg kg^−1^ soil dry weight, and experimental procedures, which lasted 15 days, were performed as previously described (Wong et al., 2005). Four hundred milligram PHE was dissolved in the 10 mL mixture of hexane: acetone (1: 1, v/v) for treating soils. Control soil treatments without heavy metals received 10 mL of sterile deionised water. Abiotic controls were prepared by adding and mixing 10 ml of 20 g L^−1^ sodium arsenite aqueous solution into soil for measuring the abiotic loss of PHE. Soil samples were placed into a fume hood overnight for the solvent volatilization. For each treatment, the soil was placed in a simulated environment with ambient temperature of 25–30°C and relative humidity of 60–90%. Moisture content of all soil treatments was maintained at relative humidity from 10 to 15% throughout the experiment. All four soil treatments were performed in duplicate, as summarized in Table S1.

#### PHE Extraction and Analysis

Residual PHE in soil samples (5 g soil, dry-weight equivalent) was prepared based on previous work (Wong et al., 2005) by using the microwave extraction technique (MCR-3, Jiecai Instrument Equipment Co., Ltd, Zhengzhou, China). Extraction parameters were set as follows: mixture of hexane: acetone (1: 1, v/v); at 700 W; 130°C; 30 min. A Hewlett Packard (HP) model 7890A−5975C gas chromatograph-mass spectrometer (GC-MS, HP, USA) was used for PHE analysis. All analysis was carried out using helium as the carrier gas. PHE standards with concentration of 40 mg mL^−1^ in the mixture of hexane/acetone (1: 1, v/v) were prepared for calibration. GC-MS operating conditions were set as follows: initial oven temperature was held at 40°C for 1 min, followed by an increase to 250°C at 10°C min^−1^. This temperature was held for 3 min and the total run time was 35 min. PHE analysis was performed on days 0, 1, 3, 5, 7, 9, 11, 13, and 15. These researchers adopt similar methods for the determination of PHE degradation rate (Crampon et al., 2014, 2017; Niepceron et al., 2014).

## Results and Discussion

### Identification and Classification of a PHE-Degrading Bacterial Strain

Strain FM-2 exhibited Gram-negative (Figure S1a), motile, short rod-like phenotypic characteristics (Figure S1b), without spores. Colonies grown on LB agar were ~1–2 mm in diameter, milky white in color, circular, integrated, convex, and transparent with intact margins (Figure S1c). Analysis of the 16S rRNA gene sequence revealed highest similarity (99%) between strain FM-2 and *B. fungorum* UFLA04-155 (GenBank accession no. GU144370; Figure S2). Physiological and biochemical characteristics of strain FM-2 are summarized in Table S2, and are shared with *Burkholderia fungorum* LMG 16225T (Coenye et al., 2001; Andreolli et al., 2011), hence the isolated strain was identified as an *B. fungorum* strain. Sequence analysis and multilocus sequence typing (MLST) of *Burkholderia* species has classified them into two main groups; *Paraburkholderia* (so-called environmental bacteria) and pathogenic species (Oren and Garrity, 2015; Dobritsa and Samadpour, 2016). However, this separation of *Burkholderia* species into beneficial (good) and detrimental (bad) categories purely based on taxonomy has been questioned (Kaur et al., 2017). Therefore, strain FM-2 was classified as genus and species *B. fungorum* and named *B. fungorum* FM-2.

### Growth and Degradation of PHE by Strain FM-2

#### Enrichment and Adaptation of Strain FM-2 During Degradation of PHE

Biodegradation of PAHs is difficult and inefficient (Haritash and Kaushik, 2009). In the present work, strain FM-2 was enriched from MM inoculated with oil-contaminated soil samples from a Xinjiang oilfield, and found to utilize PHE as a sole carbon source when incubated during 2 days under aerobic conditions (shaking at 200 rpm) at 25°C. The culture media containing the enriched FM-2 strain degrading PHE (300 mg L^−1^) turned from transparent to dark yellow (Figure S3), accompanied by a slight increase in biomass. Following several rounds of sub-culturing, a well-adapted biomass was obtained that could efficiently degrade PHE.

#### Determination of Cell Growth Curves and PHE Degradation Capability

**Figure 1:**
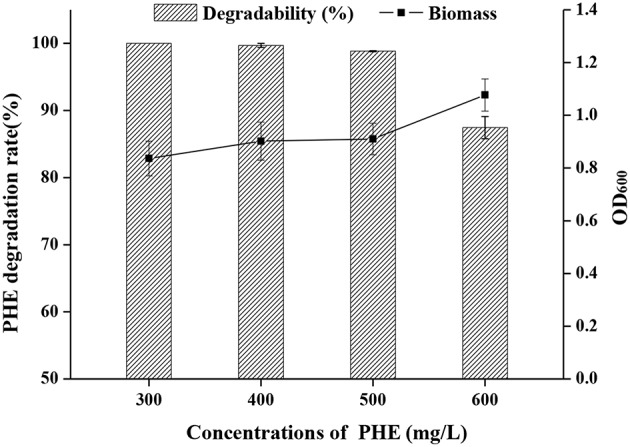
Effect of initial concentration of PHE (300–600 mg L^−1^) on a degradation rate and OD_600_ by strain FM-2.

The growth curve of strain FM-2 revealed that bioactivity was highest after the seed liquid was cultured for 48 h (Figure S4). This was therefore selected as the inoculum, and the density of the seed liquid was 10^8^ colony-forming units (CFU)/mL. PHE degradation by strain FM-2 was investigated using minimal medium supplemented with PHE ranging from 300 to 600 mg L^−1^. As shown in Figure 1, 300, 400, and 500 mg L^−1^ PHE was almost completely degraded within 3 days, while the bacterial cell density remained almost unchanged. These results indicate that *B. fungorum* FM-2 is a more powerful biodegrader of PHE than *Sphingobium chlorophenolicum* C3R that metabolizes <65% PHE within 3 days at an initial PHE concentration of 300 mg L^−1^ in a liquid culture comparable to the present work (Colombo et al., 2011). FM-2 is also superior to *Pseudomonas stutzeri* ZP2 that utilizes 88% of available PHE after 3 days in liquid culture containing 250 mg L^−1^ PHE (Zhao et al., 2009). A similar result was obtained from *Bacillus thuringiensis* FQ1 isolated from the soil of steelmaking plant in Heilongjiang Province, China. The degradation rate of PHE was 86.23% after adding PHE at 100–500 mg L^−1^ for 5 days (Jiang et al., 2015). However, Tao et al. demonstrated that the biodegradation efficiency of *Pantoea agglomerans* strain at low concentration of PHE (50 mg L^−1^) was only 78.4% after 15 days of culture, which was isolated from a petroleum-contaminated site at Karamay in Xinjiang in China (Tao et al., 2018). In addition, Janbandhu and Fulekar focused on PHE degradation by bacterial mixed culture, which reported that the microbial consortium *(Sphingobacterium* sp., *Bacillus cereus*, and *Achromobacter insolitus*) could degrade 250 mg L^−1^ of PHE, and its degradation efficiency was 56.9% after 14 days of culture (Janbandhu and Fulekar, 2011). It can be seen that strain FM-2 has obvious advantages compared with the strains mentioned above for PHE degradation.

### Optimal Cultivation Duration, Temperature, Salinity and pH for PHE Degradation

**Figure 2:**
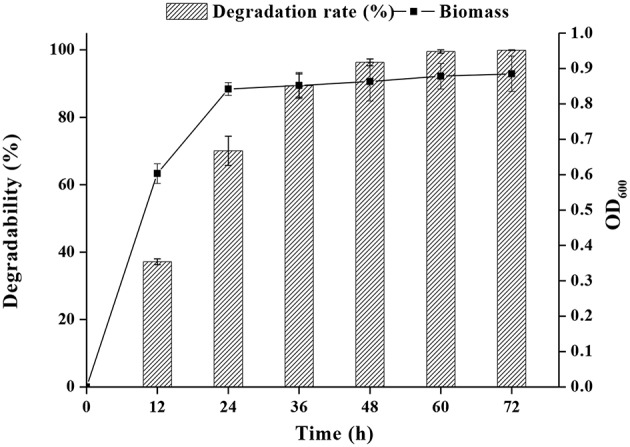
Effect of incubation time on PHE degradation at 25°C under shaking conditions (200 rpm) within 72 h.

As shown in Figure 2, both FM-2 cell growth and PHE degradation were increased after incubation for 2 days. Strain FM-2 degraded 96.31, 98.45, and 99.67% of PHE within 48, 60, and 72 h, respectively. Therefore, a culture duration of 2 days was chosen for subsequent experiments.

**Figure 3:**
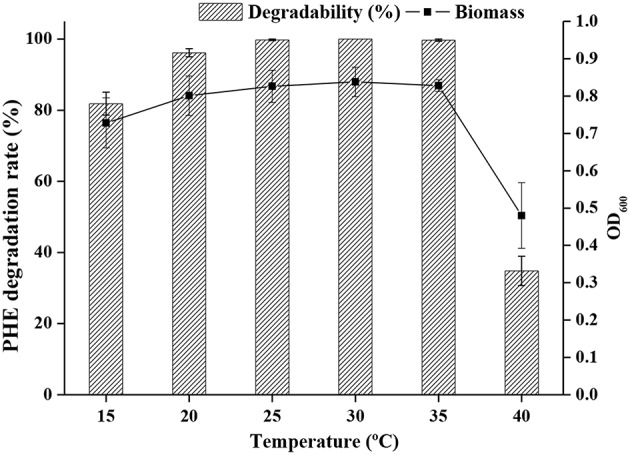
Growth of bacteria and degradation rate at different temperature.

The effect of temperature on PHE degradation was investigated, and maximum PHE degradation was observed at 25°C, followed by 30 and 35°C (Figure 3).

**Figure 4:**
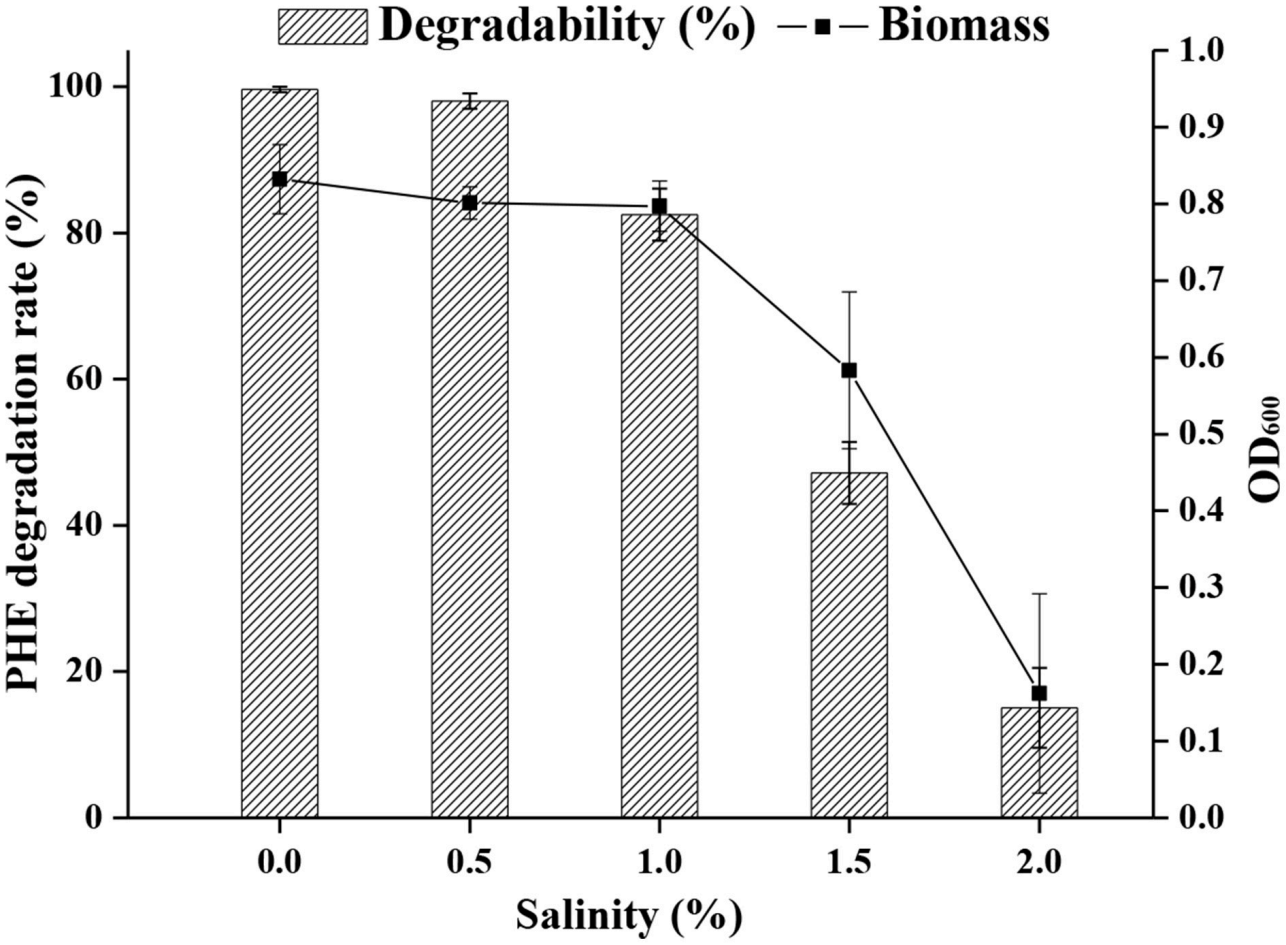
The ability of strain FM-2 to tolerate various salinities.

The optimal salinity for cell growth and PHE removal was investigated, and 0.5 % (w/v) NaCl was found to be optimal (Figure 4). A relatively high cell density was achieved in media containing up to 1% NaCl, but NaCl higher than 1% was detrimental to cell growth.

**Figure 5:**
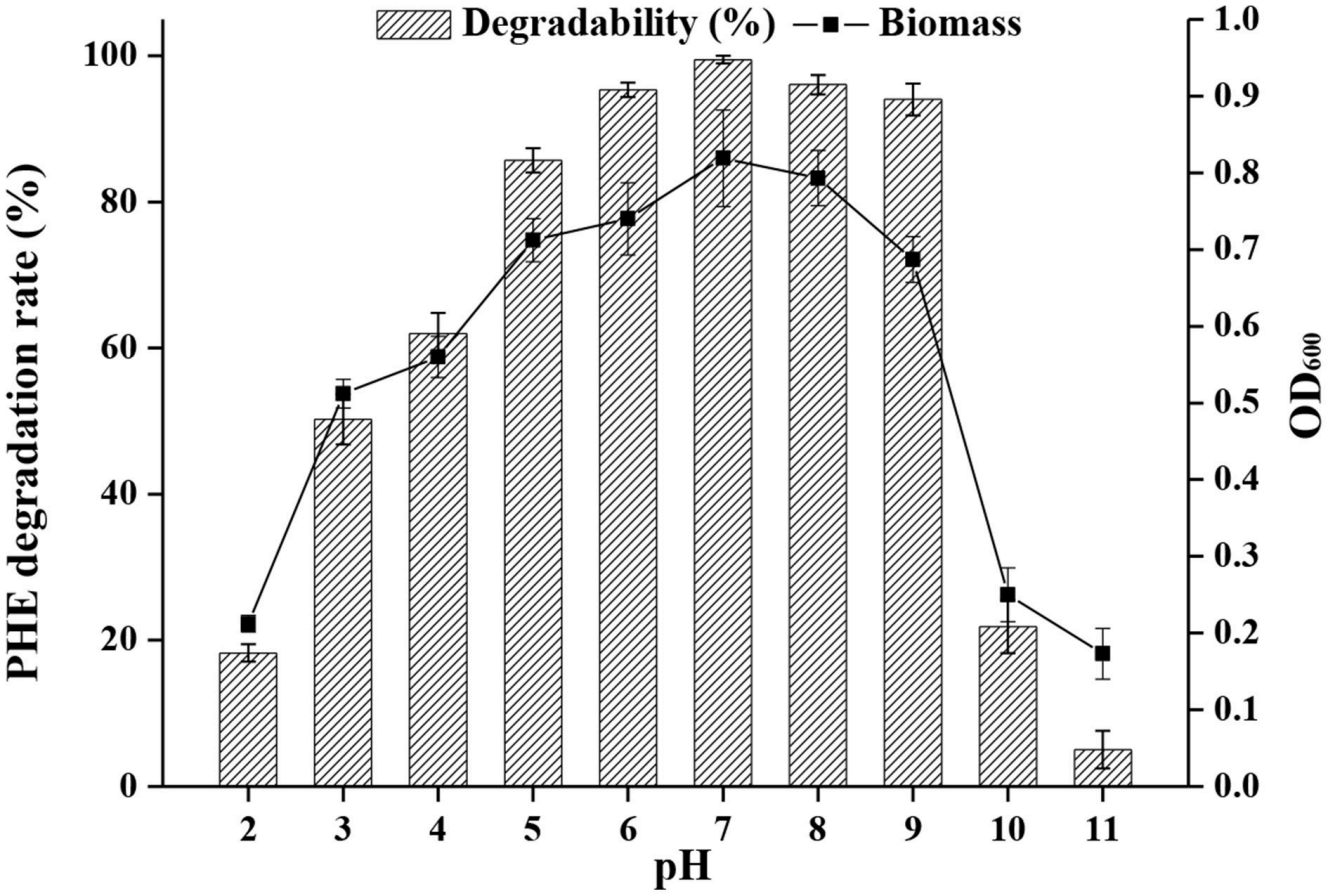
Effect of different pH at 25°C under shaking conditions of 200 rpm.

The pH of effluents from industrial processes can fluctuate widely due to their complex composition, and this represents a challenge for biodegradation by microorganisms (Alva and Peyton, 2003). The effects of pH on PAH bioremediation have been investigated (Birolli et al., 2017), and a pH range of 3.7 to 7.7 generally favors bacterial growth. Bacterial strains are remarkably sensitive to a pH <5 and >9, and removal of pollutants is generally decreased under such extreme acidic or basic conditions (Al-Thukair and Malik, 2016). However, biodegradation of PHE has been shown to occur even at pH 10 (Lin et al., 2014). Indeed, as shown in Figure 5, strain FM-2 could degrade over 94% of PHE at an initial concentration of 300 mg L^−1^ over a pH range from 6.0 to 9.0. The highest degradation rate occurred at pH 7.0, and degradation was slightly lower at pH 8.0. Furthermore, the bacterial cell density (OD_600_) was comparable at pH 7.0 and 8.0, in accordance with the PHE degradation rate, but the cell density was decreased markedly at pH 2.0, 10.0, and 11.0, indicating some limitations for cell growth. However, when the pH of the culture medium was three, the OD_600_ value of strain FM-2 was 0.512, and the PHE-degrading rate was 50.23%, demonstrating that the strain is strongly resistant to acidic conditions. This finding is important because only very few PAH-degrading bacteria such as *Mycobacterium* (Grosser et al., 1991) and *Sphingomonas* (Kästner et al., 1998) are capable of oxidizing PAHs at acidic pH. *B. fungorum* T3A13001 is reportedly active over a wide pH range, and can biodegrade 59–62% of available pyrene in liquid culture at pH 5.0 and 9.0 (Al-Thukair and Malik, 2016). This study only surveyed the degradation of pyrene at pH 5.0 and 9.0, while our current research found that PHE degradation by strain FM-2 was influenced over a broader pH range. *Burkholderia* species such as the identified FM-2 are renowned for their acid tolerance as well as their PHE catabolic potential.

### Heavy Metal Tolerance of the PHE-Degrading FM-2 Strain

Although the impact of heavy metals on microbes has received much attention (Teitzel and Parsek, 2003; Tang et al., 2006a; Abou-Shanab et al., 2007; Choudhary and Sar, 2009; Pepi et al., 2009), the impact of heavy metals on the degradation of PHE by microorganisms is poorly understood. In the present study, the free Cd ion concentration in the culture medium was 39% according to Cd-selective electrode measurements. Similarly, ion-selective electrodes in previous studies have measured free ion concentrations 20 and 48% for Pb and Zn, respectively (Ramadass et al., 2016). Given that the free metal ion concentration in the culture medium is generally less than these values, subsequent experiments were carried out with LMM medium (Ramadass et al., 2016).

**Figure 6:**
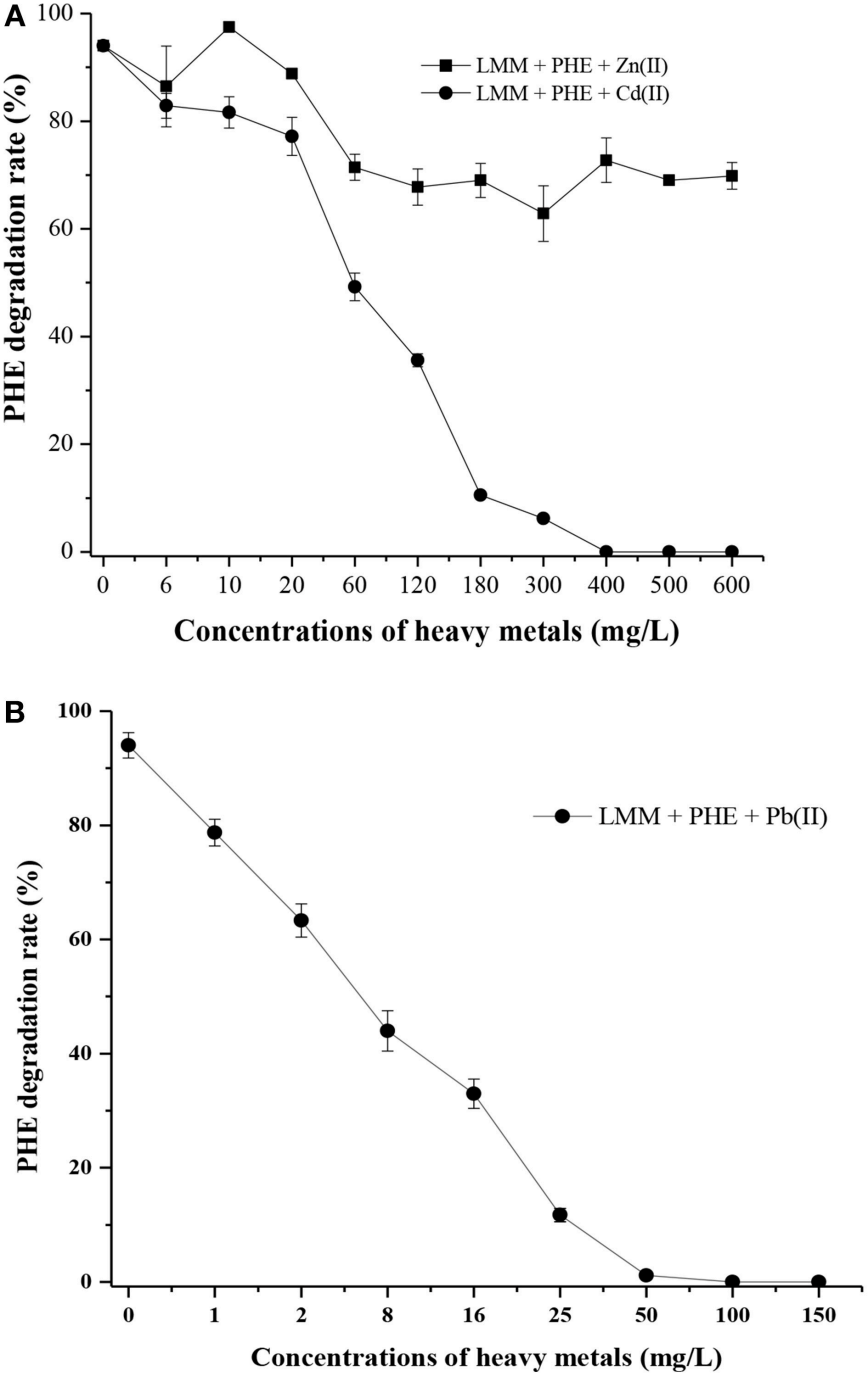
Effect of heavy metals on PHE degradation within 7 d **(A)**, Zn (II) and Cd (II); **(B)**, Pb (II).

As shown in Table S3, strain FM-2 exhibited an MIC of 400 mg L^−1^ for Cd (II) and 50 mg L^−1^ for Pb (II). However, an MIC value for Zn (II) was not obtained because strain FM-2 still displayed potent degradation of PHE at a Zn concentration of 1,200 mg L^−1^ (data not shown) after 7 days. Addition of Zn at 1,200 mg L^−1^ did not inhibit bacterial growth, possibly due to complexation of Zn (II) by bacterial biosurfactants. As shown in Figure 6A, PHE biodegradation by strain FM-2 was almost unaffected by addition of Zn (II).

When Cd (II) was added to LMM medium at low concentrations (0–20 mg L^−1^), it had little effect on PHE degradation or FM-2 cell growth. However, when the Cd (II) concentration was increased to 60 mg L^−1^, cell growth was significantly diminished, and PHE degradation was also decreased to 49.25%. As shown in Figure 6B, low concentrations (1–25 mg L^−1^) of Pb (II) in LMM medium lowered the PHE degradation rate after 7 days. Our results indicate that Pb (II) was the most toxic heavy metal and potent inhibitor of PHE degradation by strain FM-2, whereas low concentrations of Zn (II) promoted cell growth. Therefore, resistance to heavy metals was ordered Zn (II) >Cd (II) >Pb (II). This could be due to different responses to heavy metals in PHE degraders (Thavamani et al., 2015), and strain FM-2 clearly displayed maximum resistance against Zn. As Table 1 showed, strain FM-2 exhibited higher PHE degradation efficiency compared with strain species reported previously under different PHE concentrations or pH or supplement of heavy metal ions.

**Table 1:** Degradation of PHE by various bacteria/fungi.

**No**.	**Organisms**	**Initial PHE concentration (mg L^**−1**^)**	**Degradation efficiency (%)**	**Time required (days)**	**pH tolerance range**	**Heavy metal concentration**	**References**
1	*Massilia* sp.*WF1*	1	100	1/3	—	—	Gu et al., 2016
2	*Pseudomonas* sp. BZ-3	25	60–79	7	6–9	—	Lin et al., 2014
3	*Pantoea agglomerans*	50	78.4	15	—	—	Tao et al., 2018
4	*P. liquidambari B3* (endophytic fungus)	50	77.38	10	—	—	Fu et al., 2018
5	*Brevundimonas* sp. strain X08, *Alcaligenes faecalis* strain J08	50	100 >95	3.5	—	0.01, 0.1, 0.5 mM Cd^2+^	Xiao et al., 2010
6	*Sphingomonas* sp. GY2B	100	>85	2	7–9	—	Tao et al., 2007
7	*Cupriavidus* sp. MTS-7	150	100	4	—	—	Kuppusamy et al., 2016
8	*Pseudomonas* sp. &*Staphylococcus* sp.	200	100	10	—	—	Mnif et al., 2017
9	*Bacillus thuringiensis* FQ1	200–500 mg kg^−1^ (in soil)	>75	90	—	0–20 mg kg^−1^ Cd^2+^	Jiang et al., 2015
10	*Burkholderia fungorum* FM-2	300	>85.71	3	5–9	—	This research
11	*Burkholderia fungorum* FM-2	150 mg kg^−1^ (in soil)	>92.04	15	—	6 mg kg^−1^ Cd^2+^ or 10 mg kg^−1^ Zn^2+^	This research

### Analysis of Heavy Metal Bioaccumulation in FM-2

#### ICP-OES Analysis and Isotherm Study

**Figure 7:**
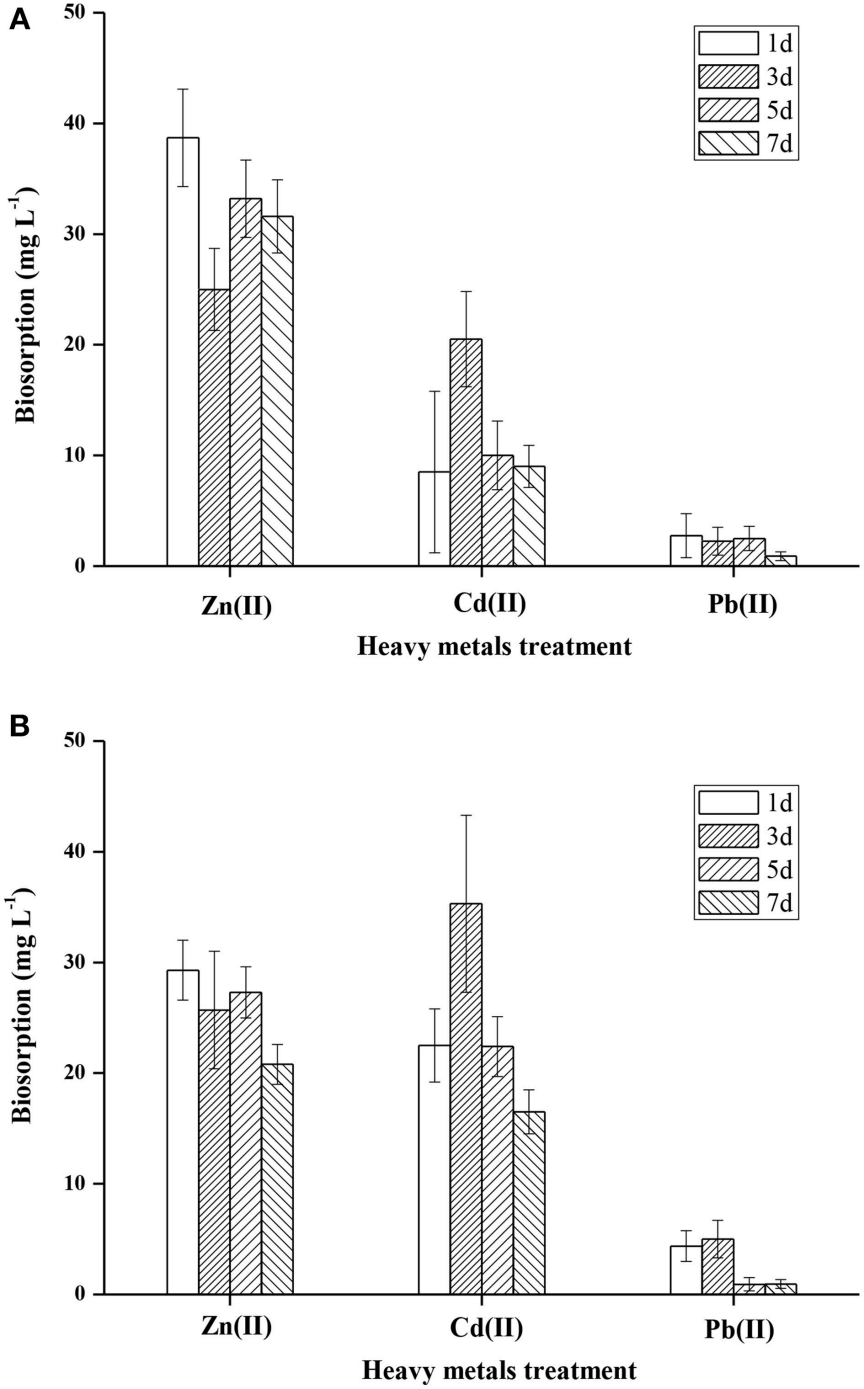
Accumulation of heavy metals in FM-2 (**A**, cell wall; **B**, intracellular space).

It is well-documented that microorganisms have a high affinity for metals, and can accumulate heavy metals by a variety of mechanisms (Irawati et al., 2017). Heavy metal accumulation in the cell wall of FM-2 was investigated, and the mean Zn (II) content was 38.7, 25, 33.2, and 31.6 mg L^−1^ after 1, 3, 5, and 7 days, respectively (Figure 7A). Thus, strain FM-2 exhibited effective bioaccumulation of Zn (II) after only 1 day of incubation at 25°C. By contrast, Cd (II) bioaccumulation peaked at 3 days at 35.3 mg L^−1^, then declined by 37 and 53% after 5 and 7 days, respectively (Figure 7B). Accumulation of Pb (II) was lowest after 5 days. Moderate amounts of Zn (II) accumulated in the intracellular space, and overall, strain FM-2 achieved high bioaccumulation of Zn (II) and Cd (II). Similarly, Durve et al. (2013) reported a slower accumulation of Pb over time in comparison with cadmium (Durve et al., 2013), and whole-cell accumulation of Cd (II) was highest in the most Cd-resistant Pseudomonas sp. (strain I10). Furthermore, intracellular Cd (II) bioaccumulation was found to be greater than that in the cell wall, demonstrating that Cd (II) bioaccumulation was related to cell growth and/or increased biomass (Chiboub et al., 2016). The Cd (II) bioaccumulation by strain FM-2 demonstrated in the present work is therefore consistent with previous reports. However, in FM-2, Zn (II) accumulation was greater in the cell wall than in the intracellular space, suggesting extrusion as a potential mechanism underpinning heavy metal resistance in FM-2 (Wei et al., 2009).

As shown in Figures S5, S6, the Langmuir and Freundlich isotherm models were used to predict the adsorption behaviors of strain FM-2 on Zn(II), Pb(II), or Cd(II). After different time of adsorption, the results led to better accordance to Langmuir model than the Freundlich model for the adsorption of Zn(II) or Pb(II), owing to higher *R*^2^ value in Langmuir model. Compared with Zn(II) or Pb(II), the result of Cd(II) fitted better in Freundlich adsorption model. The Langmuir isotherm suggests that the biosorption of metal ions can be regareded as a single-layer adsorption with no interaction between metal ions (Prasad et al., 2013).

#### SEM-EDS Analysis

Figure S7 shows SEM images of dried FM-2 biomass after Zn (II), Pb (II), and Cd (II) biosorption at a magnification of 20,000×. In the absence of metal ions, cells were short rods with smooth surfaces in a loosely-bound form (Figure S1b). However, after biosorption of Zn(II), Pb(II), and Cd(II), biomass was much reduced, and cell surfaces became rough, wrinkled, and porous. This might be due to deposition of heavy metal ions, precipitation of organic functional groups, or metal sequestration (Vishan et al., 2017a), but irrespective of the mechanism, these results suggest that heavy metal ions altered the cell morphology (Chatterjee et al., 2011) and caused significant toxicity, as shown previously for other *Burkholderia* species (Wei-hua et al., 2009). SEM images were confirmed by EDS spectra, which revealed the presence of Zn, Pb, and Cd on the cell surface.

#### FTIR Spectroscopy Analysis

FTIR spectroscopy was conducted in the range of 400–4,000 cm^−1^ to identify functional groups involved in Zn (II), Pb (II), and Cd (II) biosorption (Figures S8a–d). FTIR spectra of FM-2 biomass prepared without metal ions displayed typical absorption peaks consistent with the complex nature of bacterial biomass. Strain FM-2 exhibited a broad absorption band corresponding to hydroxyl groups between 3,500 and 3,300 cm^−1^ due to complexation of -OH groups with Zn (II), Pb (II), and Cd (II) ions. Similar results have been reported for *Bacillus cereus* after treatment with As (III) (Bahari et al., 2013; Giri et al., 2013), chromium-treated *Pseudomonas aeruginosa* (Chatterjee et al., 2011), and *Escherichia coli* after treatment of cells with Cd (II), Cr (VI), Fe (III), and Ni (II) (Abhay et al., 2016). A peak at ~2,922 cm^−1^ was assigned to stretching vibrations of C–H bonds of methylene groups (Figure S8c), in accordance with previous studies (Zhang and Min, 2009; Vishan et al., 2017b). According to reports by Chandra et al. (2014) and Abhay et al. (2016), the strong stretching vibration band at 2,361 cm^−1^ representing CO_2_ hydrates is shifted to a higher frequency. A peak was observed at ~1,634 cm^−1^, which is in 1,640–1,550 cm^−1^ region that corresponds to the bending vibrations of amines (–NH), consistent with primary and secondary amide groups of amide and protein peptide bonds. This peak was shifted to a higher frequency (~1,636 cm^−1^) following complexation of amide groups with Zn (II), Pb (II), and Cd (II) ions. The observed absorption band around 1,398 cm^−1^ could be assigned to the C = O stretching of carboxyl groups (Kazy et al., 2006), and peaks at 1,175 and 1,049 cm^−1^ may be attributed to C–N stretching vibrations of amine groups, P–O–C links of organic phosphate groups, or P–O vibrations of the (C–${\text{PO}}_{3}^{-2}$) moiety (Jiang et al., 2004). Additional small peaks were also observed at 1,003 cm^−1^ that may be attributed to substituted ethylenic CH–CH groups or glycoside bonds in the polysaccharide structure (Pagnanelli et al., 2000). The peak observed at 669 cm^−1^ in the fingerprint region was shifted to 613 cm^−1^ (in Figure S8c), suggesting the involvement of aromatic amino acids during the biosorption of heavy metals (Prasad et al., 2011). Binding of these groups to metal may form intense δ (M–O) and δ (O–M–O) bonds with stretching vibrations at 800–400 cm^−1^ (Kazy et al., 2006). These FTIR spectroscopy results strongly indicate that carboxyl, amide and phosphate groups are the dominant functional groups involved in metal ions interactions in FM-2.

### Bioremediation of Soil Co-contaminated With PHE and Heavy Metals

**Figure 8:**
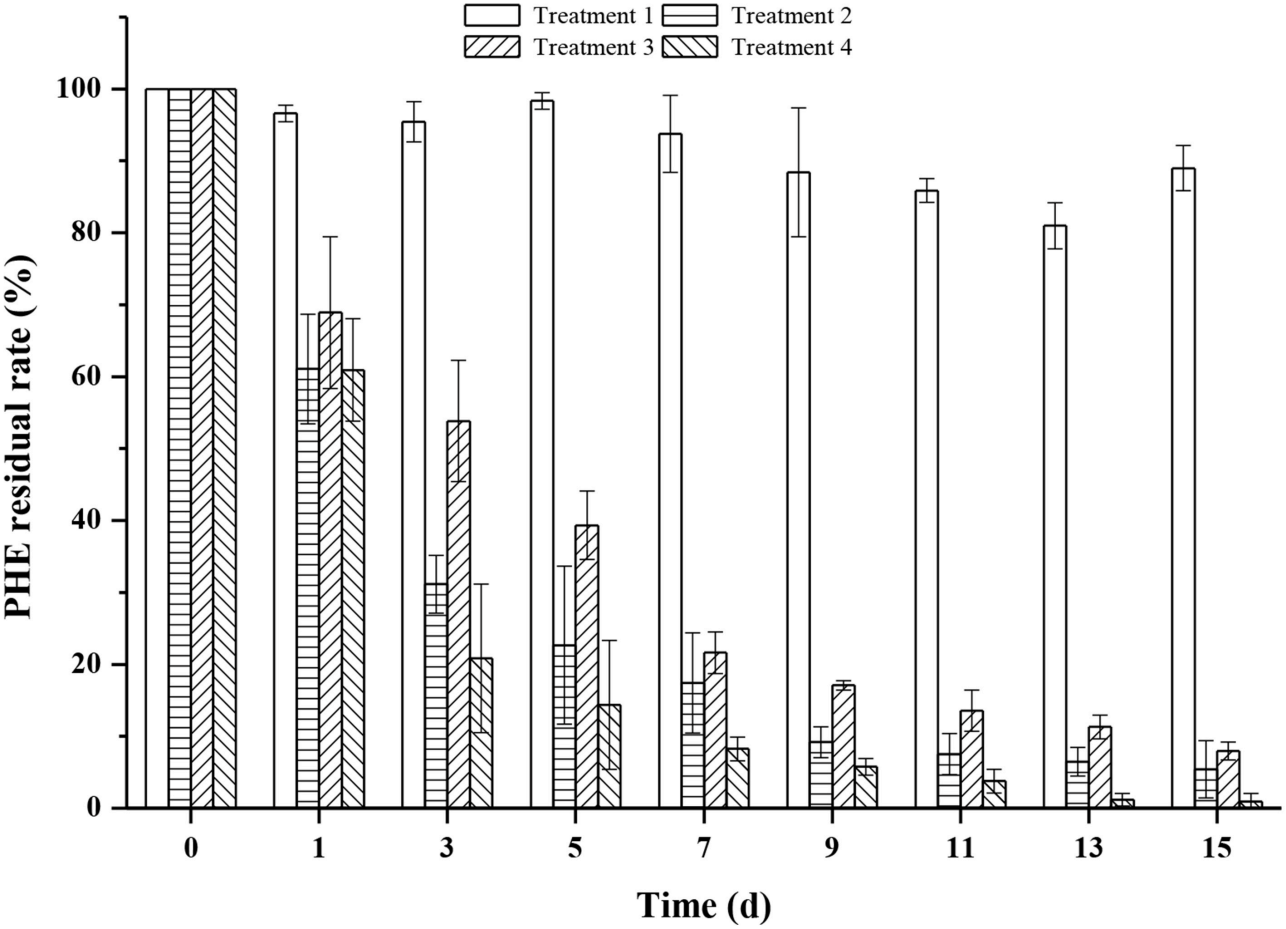
PHE residual rate (%) in co-contaminated soil (all soils had a concentration of PHE 150 mg kg^−1^ dry soil on day zero) (Treatment 1, sterilized soil + PHE; Treatment 2, sterilized soil + PHE + FM-2; Treatment 3, sterilized soil + PHE + Cd (II) + FM-2; Treatment 4, sterilized soil + PHE + Zn (II) + FM-2).

In a previous time-course metagenomic study (Kato et al., 2015), a soil sample artificially contaminated with four aromatic compounds indicated syntrophic degradation of PHE by *Mycobacterium* and *Burkholderia* (Ohtsubo et al., 2016). Illumina sequencing was performed to analyze changes in bacterial communities, and *Burkholderia* was the dominant genus in all treated soils (Guo et al., 2017; Li et al., 2017). In another study, *Alcaligenes* sp. strain J08 was found to biodegrade PHE effectively, even under 0.5 mM Cd stress conditions (Xiao et al., 2010). Moreover, addition of *Bacillus thuringiensis* FQ1 into the soils containing PHE of 300–500 mg kg^−1^ with Cd treatments, increased PHE biodegradation efficiencies that were more than 75% for 3 months (Jiang et al., 2015). Similarly, biodegradation of PHE was boosted in soils co-contaminated with 140 mg kg^−1^ zinc (Wong et al., 2005). Song et al. have reported that saponin (a plant-derived biosurfactant) could desorb PHE from contaminated soils, and it also had the potential to remove heavy metals and PAHs from heavily contaminated soils (Song et al., 2008). Given that strain FM-2 displayed a low tolerance of Pb (II), as described in section Heavy metal tolerance of the PHE-degrading FM-2 strain above, soil experiments were carried out with the optimum PHE-degrading concentrations, which were 10 mg L^−1^ for Zn (II) and 6 mg L^−1^ for Cd (II), as summarized in Table S1. Figure 8 shows that PHE was removed effectively in treatments 2, 3, and 4 after culturing for only 1 day, and the removal efficiency for treatment 2 was 90.81% after 9 days, with treatment 4 showing similar efficacy. The PHE degradation rate of treatment 4 was higher than that of treatment 2 after 3 days, which indicates that Zn (II) significantly enhanced he ability of strain FM-2 to degrade PHE, consistent with a previous report on PHE degradation in Zn-containing soils (Wong et al., 2005). However, the degradation rate was slower after 3 days in treatment 3, and reached 82.91% after 9 days. At this point, the PHE degradation rate was <7.9% that of treatment 2, and Cd (II) strongly inhibited the ability of strain FM-2 to degrade PHE. The residual rates of PHE degradation was 89% after 15 days in treatment 1 not inoculated with strain FM-2 due to natural attenuation. PHE was almost completely degraded within 11 days in treatment 4. Figure 8 shows that addition of strain FM-2 significantly increased the degradation rate of PHE in soil. Although the addition of 6 mg L^−1^ Cd (II) clearly inhibited degradation of PHE in soil, strain FM-2 still enhanced PHE degradation under these conditions, suggesting that strain FM-2 is a candidate strain for bioremediation of PHE and heavy metals. The capacity of strain FM-2 to tolerate Cd (II) and Zn (II) while degrading PHE demonstrates its feasibility for the bioremediation of soils and sediments co-contaminated with PAHs and heavy metals.

Up to now, many researches focus on genes related to PHE degradation and heavy metal removal (Beard et al., 1997; Spain and Alm, 2003; Singleton et al., 2009; Izmalkova et al., 2013; Olaniran et al., 2013; Gran-Scheuch et al., 2017). As Figure S9 shown, there are one amino acid sequence and two gene sequences of strain FM-2. Based on previous reports, we designed the primers and cloned PAHs dioxygenase gene of strain FM-2. The BLAST analysis showed that the PAHs dioxygenase gene of *B. fungorum* FM-2 had a 99% identity with PAHs dioxygenase subunit alpha of *Burkholderia* sp. (WP_030101499.1), which was initially determined the gene as PAHs dioxygenase gene. We also obtained the Protocatechuate 3,4-dioxygenase gene related to PHE degradation, which had 100% homology of the *pcaH* gene (Protocatechuate 3,4-dioxygenase beta subunit) sequence from *Paraburkholderia fungorum* strain ATCC BAA-463 (CP010027.1). The heavy metal removal genes of strain FM-2 were detected, and it has an identical function with the TonB dependent receptor family protein of *Paraburkholderia xenovorans* LB400 (CP008760.1), but their homology is only about 64%. TonB gene has been reported broardly, which generally involves in the transportation of many important substances such as iron, heme, vitamin B12, carbohydrates, and various metal elements transition (Wexler et al., 2002; Schauer et al., 2008; Lim, 2010; Porcheron et al., 2013; Anugraha, 2015). Due to the low similarity of the BLAST results, we assume that detected gene may be a novel one in the TonB family. In order to explore more functions of genes related to heavy metal removal, it will be studied by carring out whole genome sequencing of strain FM-2. We will modify partial genome sequence of strain FM-2 by gene editing technology to increase the removal efficiencies of heavy metals.

Potential environmental issues have been considered into microbial bioremediation process. Minerals in the environment, including Kaolinite and quartz, were found to be useful for PAHs degradation (Duval et al., 2002; Gong et al., 2016). A recent study also conducted: *Sphingomonas* sp. GY2B was attached on different minerals (kaolinite and quartz) that enhanced PHE degradation, which revealed the effects of different minerals on the microbial degradation for PAHs and the underlying mechanism (Gong et al., 2018). In biodegradation process, researchers also placed a high value on aerobic and anaerobic environmental conditions for microorganisms (Tang et al., 2006a; Kong et al., 2017; Himmelberg et al., 2018; Ni et al., 2018). Tang et al. performed experiments on PHE degradation under both microaerobic and anaerobic conditions in polluted marine sediments [surface layer (0–10 cm)] and measured the PHE degradation rate (Tang et al., 2006b). Combined kinetic analysis method, the study revealed two important factors of microbial biodegradation: bacterial adaptation state and presence of a adsorption phase (Tang et al., 2005). In addition, mini-bioreactor was used to simulate the actual environment conditions including pH, temperature and dissolved oxygen, which had strong influence on heavy metals removal (Tang et al., 2006a). In this study, strain FM-2 can degrade PHE (300 mg L^−1^) under both sufficient oxygen and microaerobic conditions. After 3 days, the degradation efficiencies of PHE were 100 and 69.7%, respectively. However, strain FM-2 cannot grow under anaerobic conditions and did not degrade PHE and adsorb heavy metals. We would further explore the removal efficiencies of strain FM-2 on heavy metals under different culture conditions (pH, temperature, dissolved oxygen, etc.). We also would use mini-bioreactor to simulate different external environments, and determine the optimal growth conditions of strain FM-2 for removing heavy metals, which aims to improve its efficiencies for practical applications.

## Conclusions

In the present study, strain FM-2 was enriched from oil-contaminated soil collected from an oilfield in Xinjiang, and found to degrades PHE as a sole energy and carbon source when supplied at 300 mg L^−1^ in minimal medium. Low concentrations of Zn (II) had a positive effect on PHE degradation, while low concentrations of Cd (II) had the opposite effect. However, the presence of Pb (II) strongly inhibited the PHE-degrading capability of strain FM-2. ICP-OES, SEM-EDS, and FTIR spectroscopy analyses following metal accumulation further confirmed that metal ions were associated with the cell wall and intracellular space of strain FM-2. Importantly, strain FM-2 displayed versatile catabolic activity and the ability to tolerate high concentrations of toxic metals while degrading PHE. Additionally, FM-2 exhibited high tolerance to acidic pH and very few PAH-degrading bacteria capable of oxidizing PAHs at acidic pH have been reported to date. These findings suggest that the multifunctional *B. fungorum* FM-2 strain may offer a cost-effective bioremediation strategy for soils and sediments contaminated with multiple pollutants, most notably in acidic environments suffering long-term pollution.

## Author Contributions

LH made substantial contributions to the experimental design and revised the article critically for important intellectual content. XL made substantial contributions to the acquisition of data, and the analysis and interpretation of data as well as drafting the article. XH conducted the Fourier-transform infrared spectroscopy test. YC tested the soil. WP was responsible for scanning the electron microscopy-energy and undertaking the dispersive X-ray spectroscopy test. JH was responsible for inductively coupled plasma optical emission spectroscopy test. PG conducted the phenanthrene biodegradation test.

### Conflict of Interest Statement

The authors declare that the research was conducted in the absence of any commercial or financial relationships that could be construed as a potential conflict of interest.
