# Supplementary material for: Biodegradation of Phenanthrene and Heavy Metal Removal by Acid-Tolerant Burkholderia fungorum FM-2
Source: Front Microbiol. 2019 Mar 14;10:408. doi: 10.3389/fmicb.2019.00408 (PMC6427951; doi:10.3389/fmicb.2019.00408)
Supplement: Supplementary file 1 [file Data_Sheet_1.docx]

*Supplementary Material*

**Biodegradation of** **phenanthrene and heavy metal removal by acid-tolerant** ***Burkholderia fungorum* FM-2**

Xin-xin Liu, Xin Hu, Yue Cao, Wen-jing Pang, Jin-yu Huang, Peng Guo, Lei Huang^*^

^*^Corresponding Author. College of Chemistry and Chemical Engineering, Tianjin University of Technology, Binshui West Road 391, Tianjin 300384, China. Tel.: 86-22-60214259;
*E-mail address:* [huanglei@tjut.edu.cn](mailto:huanglei@tjut.edu.cn) (L. Huang)

**Supplementary Table**

**Table S1** Parameters of Soils used for experimentation

| Treatment | 1 | 2 | 3 | 4 |
| --- | --- | --- | --- | --- |
| PHE (mg kg^-1^ dry weight soil) | 150 | 150 | 150 | 150 |
| Cadmium (mg kg^-1^ dry weight soil) | — | — | 6 | — |
| Zinc (mg kg^-1^ dry weight soil) | — | — | — | 10 |
| FM-2 (F)/ abiotic (A) | A | F | F | F |

**Table S2** Morphological and physiological characteristics of FM-2 and type strain

| Characteristic | FM-2 | *Burkholderia fungorum* LMG 16225^T^ |
| --- | --- | --- |
| Gram stain | - | - |
| Catalase activity | + | + |
| Oxidase test | + | + |
| Acid from glucose | - | - |
| Growth of 37 °C | + | + |
| Nitrate reduction | + | + |
| Bile esculin test | - | - |
| Indole production | - | - |
| Utilization of D-glucose | + | + |
| Utilization of D-Fructose | + | + |
| Utilization of D-xylose | + | + |
| Growth of 0.5%NaCl | + | + |
| Growth of 1.5%NaCl | + | + |
| Growth of 10% lactose | - | - |

+, Positive; -, Negative.

**Table S3** Tolerance of strain FM-2 to heavy metals with PHE as the sole carbon source (LMM)

| Heavy metals | Concentrations of heavy metals (mg L^-1^) | | | | | | | | | | | MIC (mg L^-1^) |
| --- | --- | --- | --- | --- | --- | --- | --- | --- | --- | --- | --- | --- |
|  | 0 | 6 | 10 | 20 | 60 | 120 | 180 | 300 | 400 | 500 | 600 |  |
| Zn | + | + | + | + | + | + | + | + | + | + | + | / |
| Cd | + | + | + | + | + | + | + | + | **—** | **—** | **—** | 400 |
|  | 0 | 1 | 2 | 8 | 16 | 25 | 50 | 100 | 150 |  |  |  |
| Pb | + | + | + | + | + | + | **—** | **—** | **—** |  |  | 50 |

+, growth; **—**, no growth
