# Supplementary material for: Biodegradation of Phenanthrene and Heavy Metal Removal by Acid-Tolerant Burkholderia fungorum FM-2
Source: Front Microbiol. 2019 Mar 14;10:408. doi: 10.3389/fmicb.2019.00408 (PMC6427951; doi:10.3389/fmicb.2019.00408)
Supplement: Supplementary file 2 [file Data_Sheet_2.docx]

*Supplementary Material*

**Biodegradation of** **phenanthrene and heavy metal removal by acid-tolerant** ***Burkholderia fungorum* FM-2**

Xin-xin Liu, Xin Hu, Yue Cao, Wen-jing Pang, Jin-yu Huang, Peng Guo, Lei Huang^*^

^*^Corresponding Author. College of Chemistry and Chemical Engineering, Tianjin University of Technology, Binshui West Road 391, Tianjin 300384, China. Tel.: 86-22-60214259;
*E-mail address:* [huanglei@tjut.edu.cn](mailto:huanglei@tjut.edu.cn) (L. Huang)

**Supplementary Figures**

**Fig. S1** Photographs of colonies and cells of strain FM-2: (a) Gram stain photograph of FM-2; (b) SEM photograph of FM-2 (20.0×k); (c) photograph of FM-2 colonies on LB solid media plate.


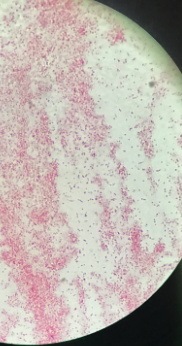



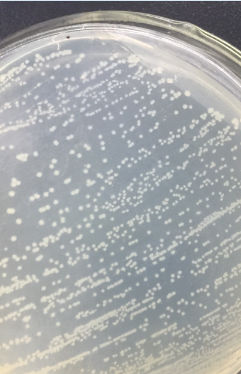


a b c

**Fig. S2** Phylogenetic analysis of strain FM-2 and related species by the Neighbor-Joining method based on 16S rRNA gene sequences. Bootstrap values (%) are indicated at the nodes, the scale bars represent 0.002 substitutions per site.

**Fig. S3** Color change of strain FM-2 using PHE as carbon and energy source caused by the formation of metabolic intermediates.


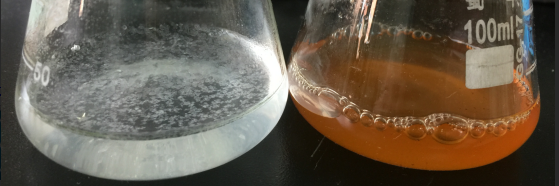


**Fig. S4** Growth curve of seed culture

**Fig. S5** Plot of Ce/Qe against the final concentration Ce (mg/L) using experimental,

Langmuir iostherms for biosorption of Zn(II), Pb(II), Cd(II), respectively, with strain FM-2.

**Fig. S6** Plot of ln(Qe) against the final concentration ln(Ce) using experimental,

Freundlich iostherms for biosorption of Zn(II) (A), Pb(II), Cd(II), respectively, with strain FM-2.

**Fig. S7** SEM-EDS of living biomass of *B. fungorum* strain FM-2, (a) living biomass after Zn (II) adsorption, (b) living biomass after Pb (II) adsorption, (c) living biomass after Cd (II) adsorption, and spectrum of EDS analysis of (a), (b) and (c).


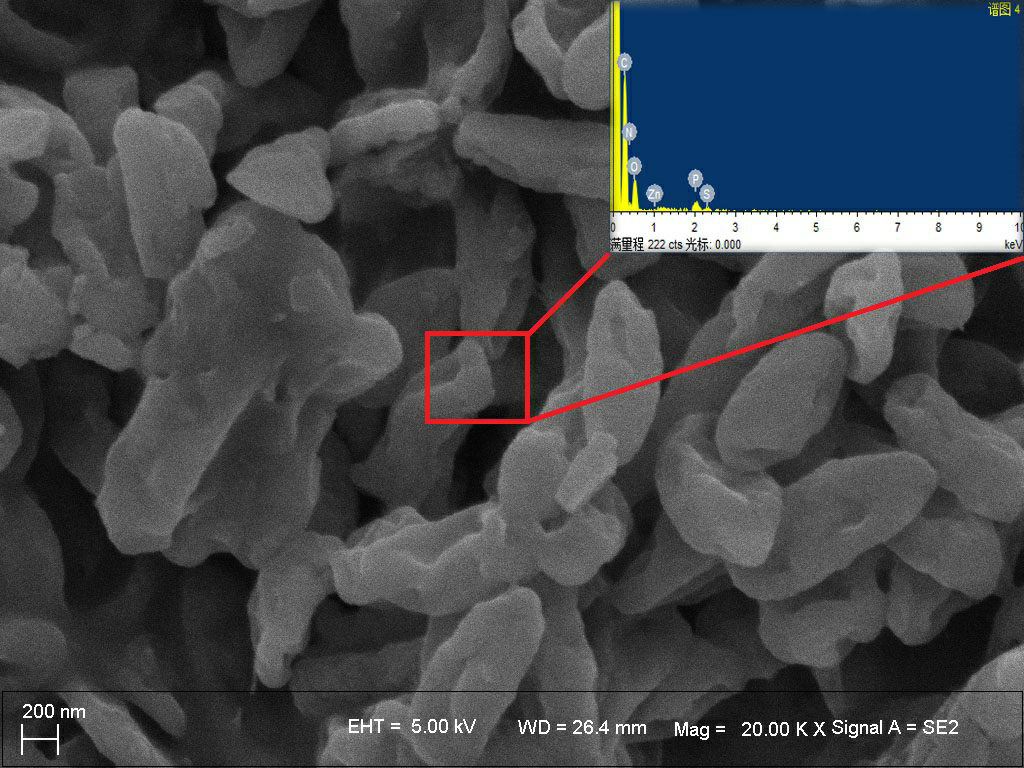


(a)


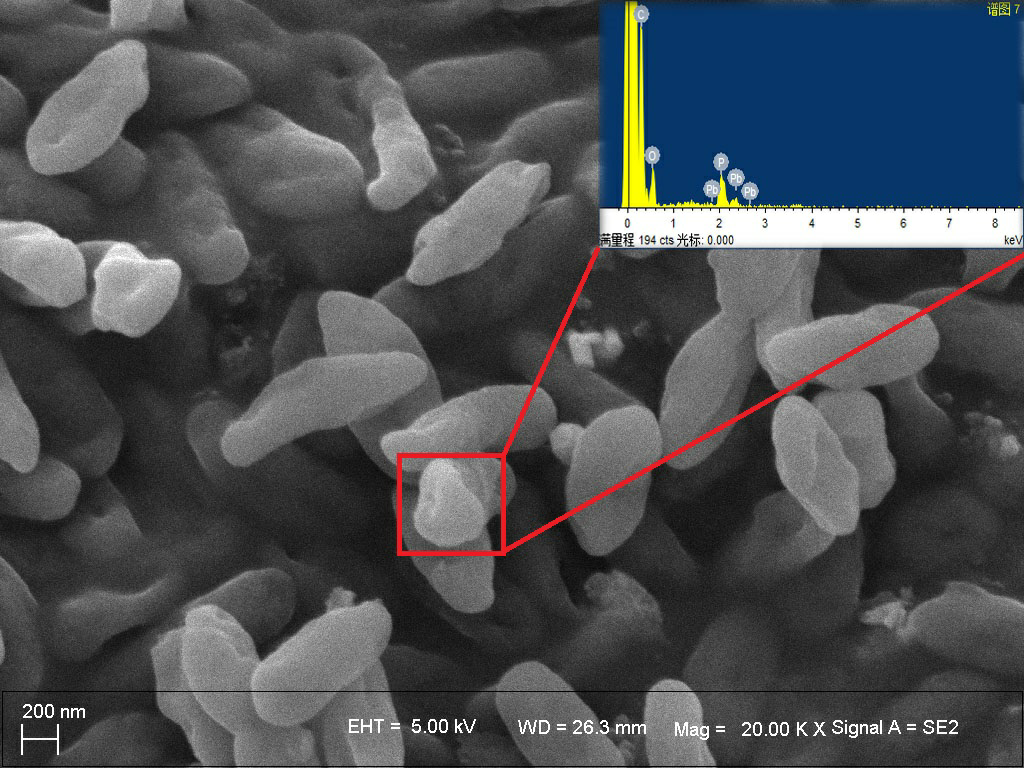


(b)


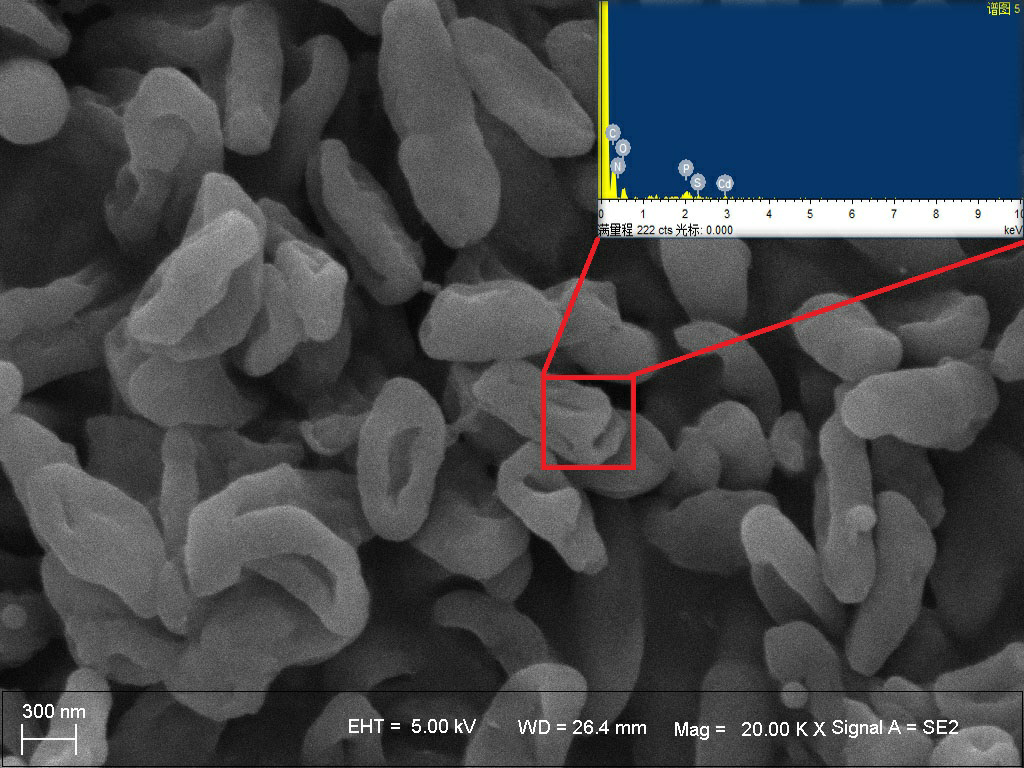


(c)

**Fig. S8** Fourier transform infrared spectroscopy (FT-IR) spectra of untreated, Zn (II)-treated, Pb (II)-treated and Cd (II)-treated FM-2 biomass in (a) 1d, (b) 3d, (c) 5d, (d) 7d.

**Fig. S9** One amino acid sequence and two gene sequences of *B. fungorum* strain FM-2, (a) Amino acid sequence of PAHs dioxygenase, (b) Gene sequence of Protocatechuate 3,4-dioxygenase, (c) Novel gene sequence of TonB family

DAYHVGWTHAASLKVLGGELSALAGNQVLPPDGAGIQITTRFGHGLGVLWNAGAAIHPPASEEGKLYRKWFDDMRPKMIQKLGESRGRLYGSHFNGTIFPNN

(a)

ATGGACGAGTCCTTTCTCACCAAGCGCGACTTCGCGTCCCACCCCGCCTA CGTTTATCCCGGCTACGGTTCGTCGGTCAAACGCGGCCCGACGCGTCCG

CTGATTCCGCTCAAAGAAAAGCTGCGCGACCAGCGCGTGCCGGTCTACGGCACCGAAGACCTCGGCGCGCTCGATCACGACCTGACGCGCAACGCGGTGCGTAATGGCGAACCGCTTGGCGAGCGCATCATCGTGACGGGCCGCGTGCTCGACGAAGGCGGCCGTCCGGTGCGCAATACGCTGGTCGAAATCTGGCAGGCCAATGCCGCCGGCCGCTATGTGCACAAGAACGATCAGCACGACGCGCCGCTCGATCCGAACTTCCTCGGCGCCGGCCGCTGCATTACCGACAACGAAGGCCGCTACCGCTTTCTGACGATCAAACCGGGCGCTTACCCCTGGGGCAATCACCCGAACGCGTGGCGTCCGAACCATATTCACTTCTCGCTGTTCGGCGACTA TTTCGGCTCACGCCTCGTCACGCAGATGTA TTTCCCCGGCGACCCGCTGC TGGCTTTCGACCCGATTTTCCAGGGCACGCCTGAACACGCCGCGAGCGCCTGATTGCAAACTTCTCGCTCGATACGACGCAAGAGGCCTATGCGCTCGGTTACGACTTCGACATCGTGCTGCGCGGCCGCAACGAAACTCCGATGGAGCGCTAA

(b)

TACGCAAGAATAGCCAGCTATCGCGGCTTCTTTAACTGTGACGATCTACGTGCGGCTCAAGCTTTGCTTGCACCGCAAACCGCCTTTCACTCTGGAGTGGCATCCCACCTCTGATATCTGCGGTGACGACCATCCGCTTTATACGACCCTCAGCGGATTCTTCCGTGTGGGTGGAACGTAACTTTTCTGAAGTCTTTGACTTTTGTGACTTATCCGCGCGTCTGTCTAGTCCCTGCCCGCTCCGGCTTCATGTCATCGGTGCCCGAACTGTTGCTGTTTGTCGCAAACCTTTTTTGGAGTTTTCTGTGTTCCGTGCTCCTGGTCAACCGCACTCAGTGACGACTAGCACCAACCCTGGGCGCAGCGCCCTCGGGGAAAGCTTTCTGAGCAACAGCATCAGTTCGTCGCTCGCGCAGATTCAATCTAATGGGCTGACGCACTAGGCCGACGCGCGACCTCTCGCATTCGTAAGATGATTCTTGTGAAGTTTCTCTCACCACATAGCAAGAGGTCCGTTACGTGTCTACCGGTGCTGACGGCCGTCACCGTGAGGCTGACAGTGCTGAGCTTATGCCTGAGTTTGAACGCGGTTTCGGCGCAGATGTTGCCTTCGATTTCACAAAACGACCAACTCTTGTATCCCGCCTATTTCGCGCTCTTGTACAACTGGAAGCCCGACGCCCCCGCCATGGCACAATCGAAATCGAGCTGCCGCCATGGTGCCCGTCAACATGATACGACAGCTCCCATTCCGCCTAATGCGAGTCTTATTACTACTCTATGGTCGTCTATATAGATCGTCGTGACCGTGATCACAGTGGTGTTCCGCGTGTTATCGTCTTGCCGCACATCTCCCTATCACCTACTGGCGGGAAGCTAAGATGCCCACACCGATGGGCCTTCTGAGTGAGTGCACTCCATTATGTGGATTGCACGCGCCGTGCACTGTCGCATTAGGC

(c)
